# GENDER DIFFERENCES IN HEALTH-RELATED BEHAVIORS OF RETIRED PEOPLE IN TAIWAN

**DOI:** 10.1093/geroni/igz038.517

**Published:** 2019-11-08

**Authors:** Nuan-Ching Huang, Susan C Hu, Ying-Wei Wang

**Affiliations:** 1 Healthy Cities Research Center, National Cheng Kung University, Tainan City, Taiwan; 2 National Cheng Kung University, Tainan City, Taiwan; 3 Health Promotion Administration, Ministry of Health and Welfare, Taipei, Taiwan

## Abstract

Many studies have indicated that lifestyles and health behaviors are important factors associated with elderly health. However, few studies have focused on such issues in Taiwan. The purposes of this study were to examine the gender differences in health-related behaviors of retired people in Taiwan. We used face-to-face interviews to collect 20 health-related behaviors among retired people aged 50-74 in Taiwan. A total of 3131 retired sample was collected including 1754 male (56.0%) and 1377 female (44.0%). The health behaviors were designed as binary variables. If the retirees executed exactly on the behavior within a month, they got 5 scores, otherwise, they got 0 scores. Then, we used factor analysis with Varimax Rotation to detect factors associated with the 20 health behaviors. Results showed that six factors were related to these 20 behaviors after conducting factor analysis. These six factors were named as 1) No tobacco, alcohol and betel nut, 2) Periodic health examination, 3) Correct medication, 4) Good habits, 5) Normal sleep and no pressure, (6) No high-fat and pickled foods. Gender differences were found in three factors: 1, 5 and 6. More female practiced every behavior in factor 1 and factor 6 than male (68.7%, 55.5% vs. 43.6%, 50.3%, p<.001, respectively). However, the male had better behaviors in factor 5 than female (54.0% vs. 47.3%, p<.001). We hope these findings could help design different health promotion programs for retirees of different genders.

